# Abuse of Non-opioid Analgesics in Germany: Prevalence and Associations Among Self-Medicated Users

**DOI:** 10.3389/fpsyt.2022.864389

**Published:** 2022-04-25

**Authors:** Christian Rauschert, Nicki-Nils Seitz, Sally Olderbak, Oliver Pogarell, Tobias Dreischulte, Ludwig Kraus

**Affiliations:** ^1^Department of Epidemiology and Diagnostics, IFT Institut für Therapieforschung, Munich, Germany; ^2^Department of Psychology, University of Arizona, Tucson, AZ, United States; ^3^Department of Psychiatry and Psychotherapy, Ludwig-Maximilians-Universität, Munich, Germany; ^4^Department of General Practice and Family Medicine, Ludwig-Maximilians-Universität, Munich, Germany; ^5^Department of Public Health Sciences, Centre for Social Research on Alcohol and Drugs, Stockholm University, Stockholm, Sweden; ^6^Institute of Psychology, ELTE Eötvös Loránd University, Budapest, Hungary

**Keywords:** non-opioid, analgesics, abuse, self-medication, epidemiological survey, over-the-counter

## Abstract

**Background:**

Abuse of non-opioid analgesics (NOA) is associated with serious health consequences. However, due to inconsistent definitions of NOA abuse, prevalence estimates for the German population are unclear.

**Objectives:**

This study aimed to estimate the 12-month prevalence of NOA abuse among self-medicated users of these drugs in the general German population and to identify risk factors.

**Methods:**

Data are from the 2015 Epidemiological Survey of Substance Abuse, a nationally representative sample with 9,204 individuals aged 18–64 years. Classification of NOA abuse was based on self-reported information according to the definition of the ICD-10-GM diagnosis F55.2 abuse of non-dependence producing substances. Multiple logistic regression was performed to examine associations between NOA abuse and sociodemographic, behavioral, and health-related variables.

**Results:**

The weighted 12-month prevalence of NOA abuse was 14.6% (95%-CI [13.2- 16.0]) among self-medicated users of these drugs. Extrapolation of the proportion of individuals abusing NOA to the German population aged 18 to 64 is 3,243,396 individuals or 6.4% (95%-CI [5.7- 7.1]). Inexplicable physical pain, being underweight, depression, hazardous alcohol use, daily smoking, illegal drug use, and frequent use of NOA (one or more times per week and daily use) were associated with an increased probability of NOA abuse. The use of cannabis was associated with a lower probability of NOA abuse.

**Conclusion:**

Abuse of NOA is highly prevalent in the German population. Against the background of increasing self-medication of NOA, healthcare providers need to be aware of potential risk factors of abuse to better identify and prevent this problem.

## Introduction

Non-opioid analgesics (NOA), like non-steroidal anti-inflammatory drugs or antipyretic analgesics such as paracetamol (i.e., acetaminophen), are the most commonly used drugs for self-medication (1). NOA are mainly used for the treatment of mild to moderate acute and chronic pain. In many countries, including Germany, NOA are available without a prescription and are so-called over-the-counter (OTC) medicines. In Germany, NOA can only be purchased in local or online pharmacies, always with the involvement of a pharmacist or a pharmacy technician (2). However, a few OTC drugs, such as vitamin supplements and herbal drugs, are available outside of pharmacies at places like drugstores or supermarkets. In Germany in 2019, 46.9% (or 706 million package units) of all drugs sold (i.e., prescription drugs, OTC pharmacy-only drugs, and drugs available at supermarkets) were OTCs available only in pharmacies. The top five most frequently sold OTC analgesics in 2019 generated a total sale of 67 million package units corresponding to 9.5% of the total sales of all OTC pharmacy-only drugs (3). Of all OTC drugs available only in pharmacies, 83.9% (or 592 million package units) were sold without a medical prescription (4). Analyses of survey data show that 21.4% of the population in Germany take one of the five most common NOA agents (i.e., aspirin, diclofenac, ibuprofen, naproxen, paracetamol) at least once a week, while 4.5% use these analgesics regularly (i.e., four or more days per week) (5).

Besides their positive analgesic effects, NOA also carry the risk of adverse events such as gastrointestinal, cardiovascular, hepatic, renal, cerebral, or pulmonary complications when used inappropriately (6–10). Results of a study of the Poisons Information Center in Erfurt Germany show that the three most frequent single drug exposures of NOA were caused by paracetamol, ibuprofen, and acetylsalicylic acid (11). The number of single drug exposures of NOA increased from 2003 to 2012 by 57% with a positive correlation between package unit sales and the frequency of exposures. Studies show that the risk of inappropriate use of these drugs is strongly enhanced by self-medication compared to prescribed use (12–14).

As NOA are easily accessible and widely used in the general population, it is important to know their potential for inappropriate use (abuse, or even dependence). In previous literature the terms more often found, and frequently used interchangeably, are *abuse* and *misuse* with several definitions existing for each. In international epidemiological studies, *abuse* is generally defined as the use of NOA for non-medical recreational purposes such as to achieve mind-altering effects (13, 15–19), while *misuse* is largely defined as the use of NOA for a legitimate medical reason but taken in a higher dose or for a period of time longer than recommended (10, 13, 15, 16, 20, 21). In other studies, *abuse* has been defined as taking a drug together with another substance, like alcohol (22), or substituting the drug with another one because the drug the individual is dependent on is not available (23). *Misuse* has also been defined as using a medicine for the treatment of symptoms other than the drug is intended (22), or when administering the drug in a manner other than recommended (e.g., intravenous administration instead of oral) (24). Thus, there is a lack of a standardized definition for the abuse and misuse of NOA. In Germany, one study on inappropriate NOA use was limited to a specific form of abuse, namely medication overuse headaches (25), and did not focus on abuse or misuse in general. Another study screened for physical and behavioral dependence on NOA in an elderly hospital population using the fourth version of the *Diagnostic and Statistical Manual of Mental Disorders* (DSM-IV) dependence criteria (26).

Neither the fourth nor the fifth version of the DSM contain a definition of NOA abuse (27, 28). However, the code F55.2 abuse of non-dependence producing substances of the ICD-10-GM coding system (*International Statistical Classification of Diseases and Related Health Problems – German Modification*) provides a definition that can be used to define NOA abuse (29). According to the ICD-10-GM code F55.2 [F55 in ICD-10-WHO (30)]:

“A wide variety of medicaments and folk remedies may be involved, but the particularly important groups are: (a) psychotropic drugs that do not produce dependence, such as antidepressants, (b) laxatives, and (c) analgesics that may be purchased without medical prescription, such as aspirin and paracetamol.

Persistent use of these substances often involves unnecessary contact with medical professionals or supporting staff and is sometimes accompanied by harmful physical effects of the substances. Attempts to dissuade or forbid the use of the substance are often met with resistance; for laxatives and analgesics, this may be in spite of warnings about (or even the development of) physical harm such as renal dysfunction or electrolyte disturbances. Although it is usually clear that the patient has a strong motivation to take the substance, dependence or withdrawal symptoms do not develop as in the case of the psychoactive substances specified in F10-F19” (29).

The authors were unable to find studies that applied the ICD-10-GM code F55.2 definition to NOA. This may be because there is no standard instrument for the ICD-10-GM diagnoses of NOA abuse that can be applied in epidemiological studies. However, a closer look at the definition of the ICD-10-GM code F55.2, if applied to NOA, reveals that the definition of abuse closely relates to four of the 11 criteria of the DSM-5 for substance use disorder (28).

Based on the ICD-10-GM F55.2 definition of abuse, the present study aimed at estimating the 12-month prevalence of NOA abuse in self-medicated users in the general German population. We also aimed to identify risk factors associated with NOA abuse, to gain knowledge on their association with NOA-related problems.

## Materials and Methods

### Study Design and Sample

Data are from the 2015 Epidemiological Survey of Substance Abuse, a population-representative cross-sectional study investigating substance use and substance use disorders among German-speaking 18 to 64-year-olds in the German population. A two-stage selection approach was applied. First, 254 sample points (i.e., cities, districts, municipalities) were randomly selected followed by a random selection of the target population using population registers. Data were collected through a standardized self-report questionnaire that was completed either on paper, through a telephone interview, or online, depending on the preference of the participant. The response rate was 52.2%. The study has been approved by the ethics committee of the German Psychological Society (DGPs; Reg.-No: GBLK06102008DGPS) and a detailed description of the methodology and design of the study can be found here: (31).

The total study sample comprised 9,204 individuals with 49.6% females, 50.4% males, and an average age of 38.3 years (*SD* = 14.7). For the present analysis, we selected individuals who used NOA in the last 12 months and reported that the NOA were not exclusively prescribed by a medical doctor. The final sample (*n* = 4,256) is described in the results section below.

### Definition of Non-opioid Analgesics Abuse

Abuse of NOA was defined based on the code F55.2 abuse of non-dependence-producing substances of the ICD-10-GM (29). For constructing the dichotomous variable of NOA abuse (yes/no) we used four questions from the Munich Composite International Diagnostic Interview (M-CIDI) that matched the ICD-10-GM definition (32, 33). Individuals that self-reported the use of NOA during the last 12 months were categorized as abusing NOA if they reported to meet one or more of the following criteria: (1) NOA were often taken in larger amounts or over a longer period than intended, (2) a persistent desire or unsuccessful efforts to cut down or control NOA use, (3) continued NOA use despite knowledge of having a persistent or recurrent physical or psychological problem that is likely to have been caused or exacerbated by the substance, or (4) craving, or a strong desire or urge to use NOA.

### Participant Characteristics and Measures

We tested sociodemographic characteristics including age (18 to 24 years, 25 to 39 years, 40 to 59 years, 60 to 64 years), gender (female, male), currently unemployed (yes, no), net household income (OECD modified equivalence scale) below the poverty threshold (yes/no), the size of their municipality [rural (< 5,000 inhabitants), small-town (5,000 to < 20,000 inhabitants), town (20,000 to < 100,000 inhabitants), city (≥ 20,000 inhabitants)], and education (low, middle, high) for association with NOA abuse. The education categories followed the International Standard Classification of Education (ISCED) which were further reduced to three groups: low (ISCED 1 and ISCED 2), middle (ISCED 3 and ISCED 4), and high (ISCED 5) (34).

We also tested behavioral and health characteristics including the following dichotomous variables (yes/no): hazardous alcohol use [i.e., a score of 8 or more on the German version of the Alcohol Use Disorder Identification Test (35–37)], daily smoking (i.e., the consumption of at least one cigarette per day during the last 30 days), consumption of cannabis in the last 12 months, consumption of other illicit drugs (i.e., amphetamines, ecstasy, LSD, heroin and other opioids, cocaine, crack, hallucinogenic mushrooms) in the last 12 months, use of opioid analgesics in the last 12 months, inexplicable physical pain in the last 12 months, and having depression in the last 12 months [screens for depression and inexplicable physical pain were taken from the M-CIDI (32, 33)]. We additionally included frequency responses on NOA use, measured for the last 30 days prior to the survey, categorized into “< one time per week,” “≥ one time per week,” and “daily use.” Based on the participants’ height and weight, we included body mass index (BMI), which was calculated according to the definition of the World Health Organization (WHO) (underweight < 18.5 kg/m^2^, normal weight 18.5 to < 25 kg/m^2^, overweight 25 to < 30 kg/m^2^, and obese ≥ 30 kg/m^2^) (38).

### Statistical Analysis

Descriptive statistics were applied to examine the sample and compare participants with and without NOA abuse. Testing for differences between the two groups was done with χ^2^ testing. To examine associations between NOA abuse and sociodemographic, behavioral, and health characteristics, we applied multiple logistic regression generating Odds Ratios (OR) and 95%-Confidence Intervals (CI) (39). The mode of administration (i.e., paper-pencil, telephone, online) was included in the model as a control variable. Interactions between NOA abuse and age, sex, education, BMI category, or daily smoking were tested for statistical significance. Inspection of Variance Inflation Factors ensured that the predictor variables were not collinear. Weights were used to account for sample differences in the distribution of age, gender, and education relative to the population, as well as their representation of the federal states and districts (40). Information on the size of Germany’s 18- to 64-year old population (*N* = 50,996,806 individuals as of 31.12.2014) (41) was used for a simple projection of the estimated prevalence to the respective total population. Data analysis was performed using Stata 15.1 SE (Stata Corp LP; College Station, TX, United States) (42). An alpha level of 0.05 was considered for statistical significance.

## Results

### Sample Characteristics

Data were available on 4,256 individuals reporting self-medication of NOA. Of these, 218 individuals were excluded due to missing information on key variables resulting in a final analytical sample of 4,038 individuals for the multiple logistic regression model. Baseline characteristics of the final sample are shown in Table 1. The majority of participants were female (*n* = 2,558; 57.1%) with a medium level of education (*n* = 1,887; 48.8%) and a mean age of 39.7 years. A total of 588 (14.6%) participants reported suffering from inexplicable physical pain and 1,436 (36.0%) individuals reported symptoms of depression.

**TABLE 1:** Sociodemographic, behavioral- and health-related characteristics of individuals using NOA and individuals included in multiple logistic regression analysis.

Characteristics	User of NOA (*n* = 4,256)	Respondents included in multiple logistic regression model (*n* = 4,038)
	
	*n* (%)	*n* (%)
Age group, years		
18 to 24	1,132 (13.2)	1,093 (13.5)
25 to 39	1,465 (35.7)	1,397 (36.0)
40 to 59	1,420 (45.7)	1,332 (45.4)
60 to 64	239 (5.4)	216 (5.2)
Mean (*SD*)	39.9 (0.2)	39.7 (0.2)
Gender		
Male	1,556 (42.6)	1,480 (42.9)
Female	2,700 (57.4)	2,558 (57.1)
ISCED		
Low	436 (11.2)	402 (10.6)
Middle	1,975 (48.6)	1,887 (48.8)
High	1,830 (40.3)	1,749 (40.6)
Not reported	15	–
Unemployed		
Yes	716 (13.0)	694 (13.0)
No	3,491 (87.0)	3,344 (87.0)
Not reported	49	–
Poverty		
Yes	475 (11.2)	434 (10.4)
No	3,781 (88.8)	3,604 (89.6)
Municipality size		
Rural*^a^*	178 (4.4)	168 (4.3)
Small town*^b^*	310 (7.4)	299 (7.6)
Town*^c^*	833 (19.7)	788 (19.8)
City*^d^*	2,935 (68.6)	2,783 (68.3)
Inexplicable physical pain		
Yes	627 (14.7)	588 (14.6)
No	3,618 (85.3)	3,450 (85.4)
Not reported	11	–
Body Mass Index (BMI)		
Underweight (< 18.5 kg/m^2^)	142 (2.3)	138 (2.4)
Normal (18.5 to < 25 kg/m^2^)	2,418 (49.7)	2,314 (50.1)
Overweight (25 to < 30 kg/m^2^)	1,129 (31.6)	1,064 (31.6)
Obese (≥ 30 kg/m^2^)	567 (16.4)	522 (15.9)
Depression		
Yes	1,519 (36.3)	1,436 (36.0)
No	2,725 (63.7)	2,602 (64.0)
Not reported	12	–
Hazardous alcohol use		
Yes	952 (19.7)	903 (19.4)
No	3,272 (80.3)	3,135 (80.6)
Not reported	32	–
Daily smoking		
Yes	652 (19.9)	601 (19.2)
No	3,574 (80.1)	3,437 (80.8)
Not reported	30	–
Illegal drug use		
Yes	96 (2.0)	89 (2.0)
No	4,150 (98.0)	3,949 (98.0)
Not reported	10	–
Cannabis use		
Yes	442 (7.6)	411 (7.3)
No	3,814 (92.4)	3,627 (92.7)
Opioid analgesic use		
Yes	134 (3.5)	123 (3.2)
No	4,096 (96.5)	3,915 (96.8)
Not reported	26	–
Frequency NOA		
< 1 time per week	3,215 (73.4)	3,095 (73.8)
≥ 1 time per week	919 (23.8)	865 (23.5)
Daily use	87 (2.9)	78 (2.7)
Not reported	35	–
NOA abuse		
Yes	569 (14.6)	523 (14.0)
No	3,669 (85.4)	3,515 (86.0)
Not reported	18	–
Mode of administration		
Telephone (CATI)	1,264 (29.0)	1,231 (30.0)
Paper-pencil (PAPI)	1,548 (38.6)	1,380 (35.9)
Internet (CAWI)	1,444 (32.4)	1,427 (34.1)

*n = observations; % = weighted prevalence rates; NOA = non-opioid analgesics; ISCED = International Standard Classification of Education; ^a^ = < 5,000 inhabitants; ^b^ = 5,000 to < 20,000 inhabitants; ^ c^ = 20,000 to < 100,000 inhabitants; ^d^ = ≥ 100,000 inhabitants.*

### Prevalence of Non-opioid Analgesics Abuse

Among all NOA users, 569 individuals met the criteria according to the ICD-10-GM F55.2 diagnosis abuse of non-dependence-producing substances. This corresponds to a weighted 12-month-prevalence of 14.6% (95%-CI [13.2- 16.0]). Extrapolating the proportion of individuals abusing NOA to the German population aged 18 to 64 years yields an estimated number of 3,243,396 individuals or a prevalence of 6.4% (95%-CI [5.7- 7.1]). Of the individuals with a diagnosis of NOA abuse 47.1% (*n* = 276) endorsed item 1 (NOA use in larger amounts or over a longer period than intended), 40.7% (*n* = 216) endorsed item 2 (persistent desire or unsuccessful efforts to cut down or control NOA use), 58.0% (*n* = 336) endorsed item 3 (continued NOA use despite knowledge of having persistent or recurrent physical or psychological problems), and 9.9% (*n* = 69) endorsed item 4 (experiencing a craving or a strong desire or urge to use NOA).

### Abuse and Non-abuse of Non-opioid Analgesics by Sociodemographic, Behavioral, and Health-Related Characteristics

Comparison of sociodemographic, behavioral, and health-related characteristics between individuals abusing NOA and those taking NOA, but not abusing them, are shown in Table 2. Individuals abusing NOA showed statistically significant higher rates of unemployment and poverty compared to non-abusers. NOA abusers were more likely to suffer from inexplicable physical pain and depression compared to non-abusers. Further individuals abusing NOA were more likely to be underweight, overweight, or obese and less likely to have a normal BMI value. Additionally, abusers of NOA reported significantly more hazardous alcohol use, daily smoking, illegal drug use, and opioid analgesic use, compared with non-NOA-abusers. Finally, participants abusing NOA also reported a significantly higher frequency of NOA use compared with non-abusers.

**TABLE 2:** Sociodemographic- behavioral- and health-related characteristics of abuser and non-abuser of non-opioid analgesics (NOA).

Characteristics	Non-abuser of NOA (*n* = 3,515) *n* (%)	Abuser of NOA (*n* = 523) *n* (%)	χ *2*	*(df)*	*p-value*
Age group, years			22.2	(3)	0.072
18 to 24	950 (13.6)	143 (12.6)			
25 to 39	1,230 (36.8)	167 (31.0)			
40 to 59	1,146 (44.4)	186 (51.3)			
60 to 64	189 (5.2)	27 (5.0)			
Gender			0.23	(1)	0.806
Male	1,299 (43.0)	181 (42.3)			
Female	2,216 (57.0)	342 (57.7)			
ISCED			19.89	(2)	0.096
Low	336 (10.2)	66 (13.5)			
Middle	1,646 (48.5)	241 (50.5)			
High	1,533 (41.3)	216 (36.0)			
Unemployed			14.96	(1)	**0.028**
Yes	587 (12.4)	107 (16.3)			
No	2,928 (87.6)	416 (83.7)			
Poverty			41.55	(1)	**0.003**
Yes	361 (9.5)	73 (15.5)			
No	3,154 (90.5)	450 (84.5)			
Municipality size			9.06	(3)	0.472
Rural*^a^*	147 (4.5)	21 (3.1)			
Small town*^b^*	264 (7.8)	35 (6.4)			
Town*^c^*	677 (19.7)	111 (20.7)			
City*^d^*	2,427 (68.1)	356 (69.8)			
Inexplicable physical pain			268.96	(1)	**0.000**
Yes	433 (12.1)	155 (29.7)			
No	3,082 (87.9)	368 (70.3)			
Body Mass Index (BMI)			34.53	(3)	**0.021**
Underweight (< 18.5 kg/m^2^)	114 (2.2)	24 (3.9)			
Normal (18.5 to < 25 kg/m^2^)	2,034 (51.1)	280 (44.1)			
Overweight (25 to < 30 kg/m^2^)	929 (31.4)	135 (32.7)			
Obese (≥ 30 kg/m^2^)	438 (15.4)	84 (19.3)			
Depression			223.21	(1)	**0.000**
Yes	1,156 (33.0)	280 (54.8)			
No	2,359 (67.0)	243 (45.2)			
Hazardous alcohol use			25.00	(1)	**0.009**
Yes	766 (18.5)	137 (24.5)			
No	2,749 (81.5)	386 (75.5)			
Daily smoking			64.31	(1)	**0.000**
Yes	494 (17.8)	107 (27.4)			
No	3,021 (82.2)	416 (72.6)			
Illegal drugs use			56.97	(1)	**0.000**
Yes	64 (1.5)	25 (4.7)			
No	3,451 (98.5)	498 (95.3)			
Cannabis use			0.11	(1)	0.845
Yes	354 (7.2)	57 (7.5)			
No	3,161 (92.8)	466 (92.5)			
Opioid analgesic use			82.85	(1)	**0.000**
Yes	87 (2.6)	36 (7.5)			
No	3,428 (97.4)	487 (92.5)			
Frequency NOA			407.24	(2)	**0.000**
< 1 time per week	2,806 (77.4)	289 (51.2)			
≥ 1 time per week	657 (20.5)	208 (41.8)			
Daily use	52 (2.0)	26 (7.0)			
Mode of administration			3.32	(2)	0.632
Telephone (CATI)	1,077 (30.1)	154 (29.4)			
Paper-pencil (PAPI)	1,190 (35.5)	190 (38.1)			
Internet (CAWI)	1,248 (34.4)	179 (32.5)			

*n = observations; % = weighted prevalence rates. NOA = non-opioid analgesics; ISCED = International Standard Classification of Education. Text in bold face denotes statistical significance with p < 0.05.*

### Factors Associated With Non-opioid Analgesics Abuse

Weighted OR and 95% CIs of the multiple logistic regression model are presented in Table 3. The following factors were associated with an increased probability for NOA abuse: inexplicable physical pain (OR = 2.13; 95%-CI [1.64− 2.77]), being underweight (OR = 2.24; 95%-CI [1.24− 4.04]), having depression in the last 12 months (OR = 1.73; 95%-CI [1.34− 2.24]), engaging in hazardous alcohol use (OR = 1.45; 95%-CI [1.09− 1.95]), engaging in daily smoking (OR = 1.34; 95%-CI [1.01− 1.79]), engaging in illegal drug use in the last 12 months (OR = 2.99; 95%-CI [1.41− 6.34]), the use of NOA more than one time per week (OR = 2.61; 95%-CI [2.00− 3.41]), and daily NOA use (OR = 3.06; 95%-CI [1.52− 6.18]). The use of cannabis was associated with a lower likelihood of NOA abuse (OR = 0.62; 95%-CI [0.39− 0.99]).

**TABLE 3:** Multiple logistic regression model for factors associated with NOA abuse among user of NOA (*n* = 4,038).

	Abuse of NOA		
	*OR*	*p-Value*	*(95% CI)*
Age group, years			
18 to 24	*Ref.*		
25 to 39	0.96	0.799	(0.70–1.32)
40 to 59	1.34	0.092	(0.95–1.88)
60 to 64	1.15	0.651	0.63–2.09)
Gender			
Male	*Ref.*		
Female	0.88	0.353	(0.68–1.15)
ISCED			
Low	*Ref.*		
Middle	1.02	0.951	(0.61–1.69)
High	1.02	0.948	(0.58–1.79)
Unemployed			
Yes	1.11	0.558	(0.82.1.86)
No	*Ref.*		
Poverty			
Yes	1.24	0.303	(0.82–1.86)
No	*Ref.*		
Municipality size			
Rural*^a^*	*Ref.*		
Small town*^b^*	1.10	0.809	(0.49–2.48)
Town*^c^*	1.71	0.102	(0.90–3.25)
City*^d^*	1.59	0.139	(0.86–2.92)
Inexplicable physical pain			
Yes	**2.13**	**0.000**	**(1.64**–**2.77)**
No	*Ref.*		
Body Mass Index (BMI)			
Underweight (< 18.5 kg/m^2^)	**2.24**	**0.008**	**(1.24**–**4.04)**
Normal (18.5 to < 25 kg/m^2^)	*Ref.*		
Overweight (25 to < 30 kg/m^2^)	1.01	0.958	(0.75–1.36)
Obese (≥ 30 kg/m^2^)	1.07	0.651	(0.79–1.46)
Depression			
Yes	**1.73**	**0.000**	**(1.34**–**2.24)**
No	*Ref.*		
Hazardous alcohol use			
Yes	**1.45**	**0.012**	**(1.09**–**1.95)**
No	*Ref.*		
Daily smoking			
Yes	**1.34**	**0.044**	**(1.01**–**1.79)**
No	*Ref.*		
Illegal drugs use			
Yes	**2.99**	**0.005**	**(1.41**–**6.34)**
No	*Ref.*		
Cannabis use			
Yes	**0.62**	**0.043**	**(0.39**–**0.99)**
No	*Ref.*		
Opioid analgesic use			
Yes	1.41	0.242	(0.79–2.53)
No	*Ref.*		
Frequency NOA			
< 1 time per week	*Ref.*		
≥ 1 time per week	**2.61**	**0.000**	**(2.00**–**3.41)**
Daily use	**3.06**	**0.002**	**(1.52**–**6.18)**
Mode of administration			
Telephone (CATI)	*Ref.*		
Paper-pencil (PAPI)	1.04	0.802	(0.76–1.43)
Internet (CAWI)	1.00	0.982	(0.73–1.35)

*OR: weighted Odds ratio; 95%-CI: 95% confidence interval; Ref.: reference category; NOA = non-opioid analgesics; ISCED = International Standard Classification of Education. Text in bold face denotes statistical significance with p < 0.05.*

## Discussion

We estimated the weighted 12-month-prevalence of NOA abuse among self-medicated users of NOA to be 14.6%, or 6.4% of the 18-64-year-old German population. Inexplicable physical pain, being underweight, having depression, engaging in hazardous alcohol use, smoking daily, using illegal drugs, and frequent use of NOA (i.e., more than once per week) were associated with NOA abuse. In contrast, the use of cannabis was associated with a lowered probability for NOA abuse.

### Previous Research on Non-opioid Analgesics Abuse

To our knowledge, only three studies have investigated the problematic use of NOA in Germany. One study investigated drug-related problems in the self-medication of OTC drugs, including OTC analgesics, identified by community pharmacists at the time the drug was dispensed (43). Findings of this study showed that the overall prevalence of drug abuse or using the drugs longer than intended was 17.1%, with analgesics the most frequently mentioned drug. The authors additionally reported that the overall prevalence of taking the wrong dosage was 6.8% (43). The second study, a review article, showed that the prevalence of medication overuse headaches (i.e., getting a headache from taking medication to treat a headache for 15 or more days per month) due to analgesics and anti-migraine agents in the general German population ranged from 0.7 to 1.0% (25).

The third study used the criteria of the DSM-IV to screen for physical and behavioral dependence of NOA in an elderly hospital population (26). According to this study, 7% (*n* = 28) of the patients fulfilled the criteria of NOA dependence. While all patients with a positive diagnosis of NOA dependence showed at least one sign of physical dependence (i.e., tolerance and/or withdrawal symptoms), most of them reported additional behavioral dependence symptoms. Of the participants diagnosed with NOA dependence, 79.2% took “the substance in larger amounts or over a longer period than intended,” 91.7% had a “persistent desire or unsuccessful efforts to cut down or control substance use,” and 25% continued to take the substance “despite knowledge of having a persistent or recurrent physical or mental problem that is likely to have been caused or exacerbated by the substance” (26, p.268).

To our knowledge, there is only one international study that has investigated NOA abuse specifically. In this study, Kouyanou, Pither (44) investigated 125 chronic pain patients attending specialized pain clinics in South London. The results showed that 48% of the patients were using NOA for pain management. According to the authors, 5.6% of the sample were diagnosed with NOA abuse (i.e., systematic use above the maximum recommended dose for more than one month), and 4% (*n* = 5) were diagnosed with NOA misuse (i.e., use often, but not systematically, and above the recommended dose) (44).

Most previous studies focused exclusively on inappropriate use of OTC analgesics (15, 45–47). However, because countries differ in terms of which analgesics are classified as OTC medicines, the possible inclusions of non-NOA drugs render comparisons with our study difficult. For example, in some countries (e.g., France, the Netherlands, Poland, the United Kingdom), codeine-combined analgesics (e.g., codeine-ibuprofen) are available as OTC (48). These opioid-containing drugs have a higher risk for abuse and dependence due to their pharmacological properties and were not considered in our study. Additionally, in some countries, like the United States, Poland, or the United Kingdom, NOA are generally easier to obtain than in Germany. For instance, in these countries, OTC analgesics can be purchased in local supermarkets or at gas stations which might create the impression to the public that these drugs are safer than prescription-only drugs, potentially resulting in higher rates of frequent and problematic use (49). Finally, the use of inconsistent definitions of abuse and misuse leads to different results in the estimation of NOA abuse between studies (19, 50). The development of standardized substance abuse terminology is urgently needed to compare prevalence estimates between countries (19).

### Risk Factors for Non-opioid Analgesics Abuse

When interpreting our findings in light of previously published work, most of the results regarding risk factors could be confirmed. We found the frequent use of NOA (i.e., more than one time per week) to be the strongest predictor of NOA abuse. Findings of a Finish study showed that frequent use of OTC analgesics is highly related to the frequency of pain symptoms, with a positive association between daily analgesic use and the frequency of pain symptoms (51). Given the fact that the frequent use of these drugs above a certain threshold has no additional positive effect, but rather increases side effects (e.g., medication overuse headache), this might lead to the abuse of NOA in the form of increasing the dose or extending the duration of intake (25, 52, 53).

In accordance with the findings of other studies, our study found daily smoking to be a risk factor for NOA abuse (5, 54, 55). One possible explanation might be that smoking causes many health problems (e.g., rheumatoid arthritis, fibromyalgia), which are associated with having more pain (5, 56–59).

In line with the findings of Abbott and Fraser (23), we found a significant association between hazardous alcohol use and NOA abuse. Wójta-Kempa and Krzyzanowski (45) reported that 26% of users of OTC analgesics consumed these drugs to cure hangovers. Additionally, epidemiological data indicate that the risk for negative health consequences, like gastrointestinal complications, more than double in the case of concurrent use of alcohol and non-steroidal anti-inflammatory drugs compared to the single use of these substances (60). This seems quite concerning considering that Germany is among the top ten countries worldwide with the highest per capita consumption of alcohol (61).

Our results showed a significant positive association between illegal drug use and NOA abuse confirming the findings of Fingleton and colleagues (17) who reported a positive correlation between illegal drug use and OTC medicine abuse in the United Kingdom. An Australian study investigating unintentional deaths attributed to the misuse of OTC analgesics identified the use of additional medications, alcohol, and illicit drugs in 80% of the cases (62).

Most interestingly, in our study cannabis use was associated with a lower probability for NOA abuse. Researchers have shown that cannabis is frequently used, in addition to NOA, for pain management (63, 64). Thus, we attribute this finding to support that, rather than take NOA for pain, participants instead consumed cannabis. Likewise, our finding of the association between inexplicable physical complaints and a higher probability for NOA abuse was not surprising and could be confirmed by previous research (65).

In line with the findings of Benotsch, Koester (18) our results indicate that depression is a strong predictor for NOA abuse. As a previous study showed, depression and chronic pain have a complex relationship with depression occurring more often in patients with chronic pain than in healthy controls (51). Additionally, studies found that some people are using NOA to treat other symptoms than pain, like stress, anxiety, sleep disturbances, and depression (23).

Finally, we found a significant association between being underweight and NOA abuse. This might be explained by the fact that being underweight is associated with chronic pain and is often comorbid with mental disorders like depression (66).

### Notes on Prevention

Early detection of problematic use of NOA is essential to avoid subsequent harm to the patient. In self-medication, pharmacists play a particularly important role as they can provide direct advice. In Germany, the S3 guideline ‘Medikamentenbezogene Störungen’ of the German Association for Psychiatry and Psychotherapy (DGPPN), in combination with a guideline on drug abuse from the German Federal Chamber of Pharmacists (BAK), provides a good orientation for healthcare providers (67, 68). According to these guidelines, detailed information on the potential risks of medications during consultation is essential. Inquiring about previous medication use, as well as pointing out that these medications should only be used for the recommended period, in addition to a referral to a medical doctor on suspicion of abuse, is essential (67). Further prevention strategies might imply careful screening of patients who receive addiction treatment or cognitive behavioral therapy for alcohol abuse, polysubstance use, or mental health concerns.

### Limitations

Our findings are subject to some limitations. All data are based on self-reported information, which is known to be prone to response biases such as underreporting or providing socially desirable answers. Another shortcoming is that with the present cross-sectional study design some population groups with increased rates of substance use are not or are insufficiently covered. This primarily concerns individuals older than 64 years, homeless people, as well as inmates or people who are accommodated in medical institutions (69). A further limitation might be concerning our definition of NOA abuse according to the ICD-10-GM diagnosis F55.2. Because, to our knowledge, there is no valid questionnaire available to assess abuse of NOA according to this definition in population surveys, we used criteria of the M-CIDI according to the DSM-5, and the ICD-10-GM definition and the DSM-5 criteria are only closely related in meaning. We also chose a conservative approach in setting the threshold to when a participant is characterized into showing abuse or not. As participants only had to fulfill one out of four criteria, it might be that some individuals met only one criterion, even though they are not abusing their drugs. This might have led to an overestimation of NOA abuse. Unfortunately, we were not able to control for individuals suffering from chronic pain (e.g., terminal cancer or rheumatologic patients) who have a permanent need for NOA use, as these variables were not included in the ESA. While the proportion of NOA use without a medical prescription among pain patients may be low, these individuals who regularly need NOA seem to have a high risk for abusing these drugs and we believe are important to include in our study.

## Conclusion

The present study provides estimates for NOA abuse in self-medicated users and the German adult population aged 18 to 64 years. Our findings indicate that there is a substantial number of individuals showing signs of abuse according to the definition of the ICD-10-GM. Against the background of increasing self-medication and the fact that abuse of these drugs is strongly associated with negative health consequences, this should be treated as a public health concern. Efforts are needed to raise public awareness about the risks of inappropriate use of these drugs to prevent subsequent harm to NOA users. Several factors were identified that increased the probability of NOA abuse. Healthcare providers should be aware of these risk factors and take early preventive action. Since pharmacists are key in preventing inappropriate use in self-medication, they should explicitly point out potential risks and side effects in consultations with customers. Finally, there is an urgent need to develop a consistent terminology regarding the use, misuse, and abuse of NOA, in addition to an accompanying operationalization and measurement instrument. These measurement tools are needed to better identify the mechanisms underlying inappropriate use of these drugs and thus better target prevention strategies at an early stage.

## Data Availability Statement

Publicly available datasets were analyzed in this study. This data can be found here: https://www.esa-survey.de/ergebnisse/datenzugang.html. Further enquiries can be directed to the corresponding author.

## Ethics Statement

The studies involving human participants were reviewed and approved by German Psychological Society (DGPs). Written informed consent for participation was not required for this study in accordance with the national legislation and the institutional requirements.

## Author Contributions

CR analyzed and interpreted the data and wrote the initial draft of the manuscript. LK designed the study. All authors gave important feedback in revising the manuscript, commented on various versions of the article, and approved the final version.

## Conflict of Interest

The authors declare that the research was conducted in the absence of any commercial or financial relationships that could be construed as a potential conflict of interest.

## Publisher’s Note

All claims expressed in this article are solely those of the authors and do not necessarily represent those of their affiliated organizations, or those of the publisher, the editors and the reviewers. Any product that may be evaluated in this article, or claim that may be made by its manufacturer, is not guaranteed or endorsed by the publisher.
